# Temporal Lobe Epilepsy and Psychiatric Comorbidity

**DOI:** 10.3389/fneur.2021.775781

**Published:** 2021-11-30

**Authors:** Valerio Vinti, Giovanni Battista Dell'Isola, Giorgia Tascini, Elisabetta Mencaroni, Giuseppe Di Cara, Pasquale Striano, Alberto Verrotti

**Affiliations:** ^1^Department of Pediatrics, University of Perugia, Perugia, Italy; ^2^Pediatric Neurology and Muscular Diseases Unit, Istituto di Ricovero e Cura a Carattere Scientifico Giannina Gaslini (IRCCS “G. Gaslini”) Institute, Genoa, Italy; ^3^Department of Neurosciences, Rehabilitation, Ophthalmology, Genetics, Maternal and Child Health, University of Genoa, Genoa, Italy

**Keywords:** temporal lobe epilepsy, psychiatric comorbidity, bi-directional hypothesis, hippocampal sclerosis, antiseizure medications (ASMs)

## Abstract

Most focal seizures originate in the temporal lobe and are commonly divided into mesial and lateral temporal epilepsy, depending upon the neuronal circuitry involved. The hallmark features of the mesial temporal epilepsy are aura, unconsciousness, and automatisms. Symptoms often overlap with the lateral temporal epilepsy. However, the latter present a less evident psychomotor arrest, frequent clones and dystonic postures, and common focal to bilateral tonic–clonic seizures. Sclerosis of the hippocampus is the most frequent cause of temporal lobe epilepsy (TLE). TLE is among all epilepsies the most frequently associated with psychiatric comorbidity. Anxiety, depression, and interictal dysphoria are recurrent psychiatric disorders in pediatric patients with TLE. In addition, these alterations are often combined with cognitive, learning, and behavioral impairment. These comorbidities occur more frequently in TLE with hippocampal sclerosis and with pharmacoresistance. According to the bidirectional hypothesis, the close relationship between TLE and psychiatric features should lead to considering common pathophysiology underlying these disorders. Psychiatric comorbidities considerably reduce the quality of life of these children and their families. Thus, early detection and appropriate management and therapeutic strategies could improve the prognosis of these patients. The aim of this review is to analyze TLE correlation with psychiatric disorders and its underlying conditions.

## Introduction

**Figure 1:**
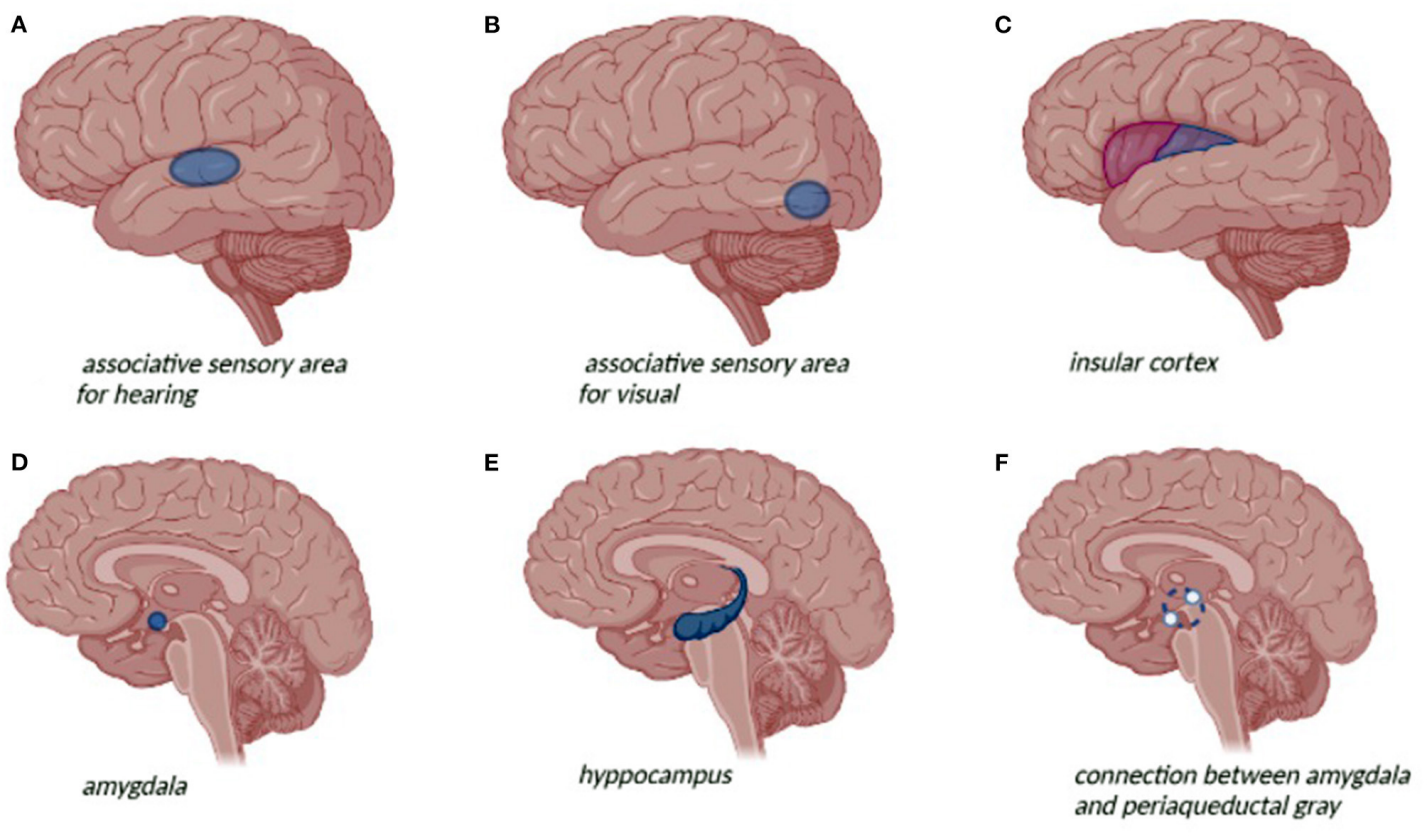
Neuroanatomical representation of the temporal lobe and related seizure symptoms and psychiatric symptoms: **(A)** auditive hallucinations; **(B)** visual hallucination; **(C)** visceral-sensory or autonomic symptom; **(D)** depression; **(E)** anxiety disorders, depression; **(F)** avoidance behavior and fear response.

Temporal lobe epilepsy (TLE) is the most common focal epilepsy (1). According to the International League Against Epilepsy (ILAE) classification (2), TLE can be divided into mesial temporal lobe epilepsy (mTLE) and lateral or neocortical temporal lobe epilepsy (nTLE). mTLE is the most common subtype and seizures originate from the hippocampus, entorhinal cortex, amygdala, and parahippocampal gyrus. The brain structures involved in nTLE are temporal neocortex that includes the superior, medial, and inferior temporal circumvolutions, the temporal-occipital and temporal-parietal junctions and the associative sensorial areas for hearing, visual, and language functions (Figure 1) (3, 4). The age at seizure onset for mTLE is lower than nTLE (10.9 years and 23.2, respectively). A personal history of febrile convulsion is more frequent in mTLE (1). Patients with TLE generally present focal impaired awareness seizures mainly characterized by the loss of consciousness associated with stereotyped automatisms, language alterations, and unilateral dystonic posturing. Focal impaired awareness seizure is frequently preceded by epileptic auras or focal aware seizures featured by visceral-sensory or autonomic symptoms and cognitive or emotional manifestations. Epigastric aura is considered a typical feature of mTLE (1, 3, 5). Dystonic posturing seems to be associated with basal ganglia involvement, whereas oral automatisms seem to be related to amygdala activation (1, 5–9). Ictal-electroencephalography (EEG) in mTLE is characterized by focal rhythmic activity in the theta range (5–9 Hz) with maximum amplitude in the basal temporal electrodes, preceded or not by bilateral hypersynchronous slowdown. nTLE might have a wider distribution at seizure onset with the typical presence of polymorphic activity at 2–5 Hz in inferior-temporal regions. Interictal-EEG in mTLE shows more frequently unilateral spike-wave, normally located in the anterior temporal region. Paroxystic discharges in medium or posterior-temporal derivations are more commonly found in the interictal-EEG records of nTLE (4). In addition to neurological features, TLE is frequently associated with psychiatric comorbidity. The prevalence of psychiatric disorders in patients with TLE is higher than in generalized epilepsy particularly in the pediatric population (79 vs. 47%) (10). Anxiety, depression, and interictal dysphoria often combined with cognitive, learning, and behavioral impairment are recurrent psychiatric disorders in the pediatric patients with TLE. Mesial temporal sclerosis (MTS) is the most common structural abnormality associated with TLE. MTS is correlated to a higher prevalence of psychiatric symptoms up to 70% in pharmacoresistant forms of TLE (11). The aim of this review is to analyze the psychiatric comorbidity of TLE and identify actual management and therapeutic perspectives that might increase the quality of life of these patients.

## Neurological Features

Temporal lobe epilepsy seizures are characterized mainly by behavior arrest and impaired awareness. Focal aware seizures are frequently reported prior to seizure onset. Autonomic or visceral-sensory seizure, characterized by the abdominal or epigastric rising sensation, is more commonly present in mTLE. Other autonomic manifestations are pallor, flushing, cyanosis, alterations in cardiac frequency and rhythm, vomiting, urinary urgency, piloerection or pupillary alterations (3, 4, 12–15). Cognitive and emotional seizures are peculiar of TLE and are characterized by I) dysmnesic features such as déjà vu or jamais vu, II) cognoscitive features with a misperception of internal or external reality, III) emotional features such as panic attacks and behavioral changes, IV) illusions and hallucinations that might set visual, auditive, or olfactory auras (3, 12, 14). Approximately 70% of focal aware seizures are followed by the impaired awareness associated with various clinical features (3). Focal to bilateral tonic-clonic seizures may occur in about 60% of patients with TLE (4). Oroalimentary automatisms and manual stereotypes movements are described in 40–80% of TLE. Particularly, oral alimentary automatism can be associated with amygdala and anterior temporal region involvement (3, 12, 16). Ictal speech, characterized by intelligible, well-articulated, linguistically correct language during crisis, is observed especially in non-dominant TLE, whereas in dominant TLE is more frequent to observe ictal aphasia and verbal automatisms (3). Unilateral dystonic posturing is more common in mTLE (1). The head deviation is common but not exclusive of TLE with supra-Sylvian structures and frontal lobe involvement (14, 17, 18). Postictal period is commonly present with a lower frequency in nTLE compared with mTLE (23.5 vs. 85%) and it is characterized by a confusional state sometimes combined with language alterations or psychiatric symptoms (4, 19).

## Psychiatric Comorbidity

Psychiatric symptoms are a common comorbidity of epilepsy (20) especially of TLE occurring in more than half of the cases. However, these manifestations are often misunderstood with a consequent reduction of quality of life of the patients. While ictal disorders are directly related to seizures, interictal psychiatric disorders may occur independently in the context of epilepsy (21). Filho et al. (22) in a study conducted on 170 patients with mTLE detected mood disorders in 25.8% of cases followed by the psychotic disorders (15.8%) and anxiety disorders (14.1%). Whereas, according to Ertem et al. (23), anxiety disorders resulted the most common psychiatric comorbidity (23%), followed by mood disorders (17%), psychotic disorders (13%), and somatoform disorders (3%). Seizures worsening and the increasing of seizure frequency are risk factors for psychopathology. Polytherapy with antiseizure medications (ASMs) has been associated with a major risk of behavioral and emotional features. Moreover, familiarity with psychiatric disorders and family disruption are predictors of psychopathology. Hippocampal sclerosis can be associated with an increased risk of frontotemporal network dysfunction resulting in the psychiatric comorbidity. Psychiatric symptoms can occur before and after seizure. Premonitory symptoms, occurring at least 30 min before a seizure, are frequently described as irritability, depression, headache, “euphoria,” and confusion (19). About 44% of children with TLE are considered at-risk for depression, while 22% are considered in the clinical significative range (24). Depression and TLE are supposed to have similar physiopathology with common involvement of hippocampus, amygdala, and long-range frontal lobe projections (24–28). The amygdala is determinant in the experience of fear and its autonomic and endocrine responses. Instead, the connection between the amygdala and periaqueductal gray is implicated mainly in avoidance behavior and fear responses. The hippocampus is important in the re-experiencing of fear. Activation of fear circuits is a major hypothesis for explaining symptoms in anxiety disorders (29–31). MRI volumetric studies have found decreased volumes of the amygdala and hippocampus in the recurrent and chronic untreated depression (26, 32). Increasing amygdala volumes, particularly on the left side, are associated with depression severity among patients with TLE (33–36). These findings may be due to enhanced regional blood flow and vascular volume as detected by the positron emission tomography (PET) (37) or secondary to dendritic remodeling with increased branching of amygdaloid neurons (38). Patients with TLE and aggressive episodes, such as interictal dysphoric depression manifestation, had a decrease of gray matter mainly in the left frontal lobe (39). Attention deficit hyperactivity disorder (ADHD) is a common comorbidity of childhood epilepsy, but the neuroanatomical correlation of ADHD with epilepsy has yet to be comprehensively characterized. High frequency of seizures and nocturnal crisis may alter attention during the day and worsen ADHD symptoms. In patients with TLE, alterations in attentional control may be charged to structural abnormalities outside the temporal lobe involving frontostriatal connections (40). The greatest deficits appear in divided attention, selective attention, and set shifting that requires a high level of processing resources. In contrast, sustained attention is less compromised and dual-task performance appears to be normal in the patients with TLE (41). Cognitive and behavioral disorders occur in almost 50% of patients with dysphoric symptoms and usually begin within 24–72 h postictally. Psychosis is less frequent (2–6%) and usually follows clusters of focal impaired awareness seizures. This disorder continues for 9–10 days on average postictally and sometimes it can last up to 3 months (19). Autism spectrum disorders (ASDs) are frequent comorbidities in childhood and adolescent epilepsies. According to Chez et al. 60.7% of children with ASD present epileptiform activity in sleep frequently localized over the right temporal region (42). EEG abnormalities appear to be more frequent in regressive autism (43). The ILAE estimates an overall prevalence of ASD in the epileptic population of ~20%, whereas the prevalence in the general pediatric population is ~1% (43, 44). The prevalence of ASD is the highest in cases with epilepsy accompanied by intellectual disability (45, 46). Children with ASD and epilepsy have greater motor difficulties, developmental delays, and behavioral problems than ASD cases without epilepsy (47). The causes of ASD are extremely variable and are sometimes common to epilepsy (48). Increasing evidence suggests that common genetic abnormalities may be associated with both epilepsy and autism (49, 50). Alterations in the mTOR pathway may lead to hyperplasticity and contribute to expression of the epilepsy-ASD comorbidity (51). According to Keown et al. (52) the enhanced local visual processing in the autistic patients may be due to the enhanced local connectivity in primary visual and extrastriate cortices, extending into the temporal lobe. Hyperconnectivity of the mesial temporal lobe has as well-been described in TLE (53–55). A diagnosis challenge is represented by psychogenic non-epileptic seizures (PNES), which are conversion disorder that is often misdiagnosed in 5–33% of patients considered affected by refractory epilepsy (56, 57). PNES mainly affect adults but can also occur in children, especially in patients with a history of multiple psychiatric diagnoses (58–60).

## From Classic Paradigm to Bidirectional Hypothesis

For several years, the “classic paradigm” explained the correlation between neurobehavioral comorbidity and epilepsy using lesion-related model (61). Indeed, according to this model, epilepsy comorbidity should be consequent to the epilepsy syndrome and its characteristics (etiology, onset, frequency, and treatment). The improvement in cognitive, neuroimaging, and clinical research highlighted neurobehavioral features heterogeneity leading to a challenge to the classic paradigm (62). For example, executive dysfunction, long considered as a pathognomonic feature of the frontal lobe epilepsy, has also been highlighted in the forms of TLE. Indeed, this feature is related to neurobiological influences exerted directly by the frontal lobe and/or indirectly through broader network connectivity (63–67). Neuroimaging studies conducted in patients with TLE showed different abnormalities involving networks disruption far from the primary seizure generation area. Particularly in patients with drug-resistant epilepsy, early-onset seizures can alter white matter development especially involving frontotemporal connections. Long disease durations in focal epilepsy syndromes such as TLE, can also lead to widespread age-accelerate cortical thinning, compounding global cognitive, memory, and processing speed impairments (68, 69). Different studies reported that psychiatric features were often present before the epilepsy diagnosis and sometimes they predate seizures onset (70–74). Other studies demonstrated that psychiatric diagnoses might occur before and after epilepsy onset with similar frequency (58, 75, 76). These findings are in contrast with the classic paradigm assuming that neurobehavioral risk increase over epilepsy development and points out the necessity to explore other common pathways (77). According to the “bi-directional hypothesis,” behavioral disorders and psychiatric features may be considered not only comorbidity but related to the same pathophysiology of epilepsy (78–83). For instance, temporal lobe dysfunction has been related to mood and anxiety disorders (84, 85). Alteration of pathways involved in temporal lobe connections was found in suicide-related behavior and post-traumatic stress disorder (29, 86). Moreover, depression in youth with TLE has a negative impact on epilepsy management and on the overall quality of life due to poor-treatment adherence, different response and tolerance of ASM, and a higher rate of hospitalization and mortality risk (81, 87–90). The close and complex relationship between epilepsy and psychiatric features should lead to considering these conditions not as an individual but in relation to each other (19, 58, 70, 72, 81, 91–93). The finding of cognitive, behavioral, and brain-imaging alterations in relatives of patients with epilepsy confirms the importance of genetic study not only in the definition of epilepsy but also of associated neuropsychiatric comorbidities (94–98). To date, studies on the impact of genetics on neurobehavioral comorbidities are scarce and limited to syndromic forms of epilepsy (99). In contrast, the study of this correlation in idiopathic forms of epilepsy remains unexplored. In a study of patients with refractory epilepsy, polymorphisms in the brain-derived neurotrophic factor (BDNF) gene were associated with depression, whereas alterations in the catechol-omethyltransferase (COMT) gene were associated with anxiety disorders (100). In addition, epigenomic, transcriptomic, and proteomic alterations certainly play a role in the development of mostly unknown neurocognitive comorbidities (62). Environmental factors are also known to be associated with neurobehavioral disorders. The stigma of epilepsy and its prevalence in poorer and less educated settings with under resourced medical services certainly plays a major role in the development of psychiatric comorbidities and overall quality of life (62). Indeed, a higher cognitive level seems to have a protective role against the neurocognitive comorbidities of epilepsy (101). Intervention toward a better lifestyle through education and psychosocial therapy can contribute to the treatment of epilepsy comorbidities. This approach should be implemented in the clinical practice and tailored to the patient epilepsy and neuropsychiatric comorbidities (62).

## Diagnosis and Pharmacological Management of Psychiatric Comorbidity

Early recognition of psychiatric features is the first step to guarantee the correct management of this disorder. It is necessary to exclude that symptoms are a consequence of seizures or an adverse effect of ASM. For the most ASMs, remarkably few studies providing robust data on the psychiatric adverse effects in epileptic patients were identified. Barbiturates, Topiramate, Valproate, and Zonisamide have been reported to cause worsening attention (102, 103). Phenobarbital, vigabatrin, zonisamide, topiramate, and levetiracetam could be associated with depression (102). When this is suspected, modification or discontinuation of the responsible drug is recommended. Instead, moderate–severe forms of psychiatric comorbidity not related to ASM require early treatment (60). A good seizure control in these patients is essential to improve psychiatric symptoms (60). No large, double-blind, placebo-controlled trials of medications for ADHD were conducted in children with epilepsy. Methylphenidate was used in small trials in children affected by epilepsy and ADHD with an improvement in attention without worsening of seizures (104–108). Amphetamine and atomoxetine may reduce ADHD symptoms without increase in seizure frequency, but little data have been collected for these treatments (109). Anxiety and depression treatment is a combination of education, psychotherapy, and medication. Cognitive behavioral therapy and medication are recommended by the American Academy of Child and Adolescent Psychiatry guidelines for moderate-to-severe disorder from Bernstein and Shaw (110). The first choice treatment is serotonin reuptake inhibitors (SSRIs) (111). No controlled trials of SSRIs are conducted in children with epilepsy, but studies on fluoxetine and sertraline have shown improvement in anxiety and depression symptoms without adverse effect on the seizure management (112). Tricyclic antidepressants are not effective in children with anxiety and depression, contrary at high dose may reduce seizures threshold with worse control of epilepsy. Bupropion and clomipramine have been associated with an increased risk of seizures (113). Treatment of autism spectrum disorder includes educational and behavioral interventions, but a pharmacological approach can be necessary for specific symptoms (114, 115). Indeed, pharmacologic treatment is warranted in the autistic patients with epilepsy. In children with ASD who have mood disorders, choosing a medication that also has mood-leveling properties may be useful. Whether there is any benefit in treating a child with ASD and epileptiform discharges in the absence of epilepsy remains debated (44).

## Surgical Approach

Temporal lobe epilepsy surgery is currently considered safe and successful in the patients with drug-resistant epilepsy. High-resolution magnetic resonance techniques and advances in microsurgery ability have led to the improvement of this therapeutic approach. However, an adequate patient selection with invasive and non-invasive monitoring including psychiatric assessment is necessary (116, 117). Resection strategies have been enhanced over time. Initially, anterior temporal lobectomy (ATL) was considered first-line treatment for mTLE. Subsequently, transsylvian Amygdalo–Hippocampectomy (AH) replaced ATL thanks to improved epileptogenic focus identification technics. Instead, patients with nTLE are treated with lateral lesionectomy without ATL with increasingly limited resections. A satisfactory outcome (Engel I/II) is achieved in 84.7% of patients treated with surgery. Lateral lesionectomy has the highest success rate (94.1%), while AH has the lowest (78.8%). This result can be related to the multiple comorbidities in children with mTLE. Indeed, patients with mTLE are less likely to achieve seizure freedom independently of the resection strategies (116). The presence of the psychiatric disorders before the surgery predisposes to psychiatric pathology at 2-year follow-up and is not correlated with epilepsy outcome. After microsurgery, anxiety and depressive disorders decreased, and psychotic disorders increase without statistical significance. *De novo* psychiatric disorders occurring after surgery represented 52% of the postoperative psychiatric pathology, 62% being psychotic disorders. These disorders became more frequent from the first year after surgery, occurring mainly in the patients seizure free (118).

## Conclusion

Psychiatric features and TLE are closely related with overlapping risk factors and common etiologies. Alterations and neurotransmission disturbances among critical networks with impaired or aberrant plastic changes might predispose patients with TLE to the development of these symptoms. Mood and anxiety disorders are the most frequent in the pediatric population. Early diagnosis of these disorders can result in better management, ensuring a multidisciplinary approach and, if necessary, appropriate treatment with increased quality of life. Routine psychological and/or psychiatric evaluation should be standard in the comprehensive care of children with epilepsy. Any comorbid psychiatric disorder must be considered in the choice of ASM, taking into consideration their potential positive and negative psychotropic properties.

Finally, advances observed in the neurosurgery particularly in resection strategies have led to additional therapeutic options. The surgical approach, besides improving neurological symptoms, can lead to a reduction in psychiatric comorbidities in these patients.

## Author Contributions

VV and GD put forward the conception of the review and wrote the manuscript. AV and EM participated in the proposal of the concept and revised the manuscript. GT, GC, and PS proposed suggestions for revision. All the authors approved the submitted version.

## Conflict of Interest

The authors declare that the research was conducted in the absence of any commercial or financial relationships that could be construed as a potential conflict of interest.

## Publisher's Note

All claims expressed in this article are solely those of the authors and do not necessarily represent those of their affiliated organizations, or those of the publisher, the editors and the reviewers. Any product that may be evaluated in this article, or claim that may be made by its manufacturer, is not guaranteed or endorsed by the publisher.
